# New Insights into Cancer Targeted Therapy: Nodal and Cripto-1 as Attractive Candidates

**DOI:** 10.3390/ijms22157838

**Published:** 2021-07-22

**Authors:** Paola Arboretto, Michele Cillo, Antonio Leonardi

**Affiliations:** Department of Molecular Medicine and Medical Biotechnology, University of Naples Federico II, Via Pansini 5, 80131 Naples, Italy; paola.arboretto@unina.it (P.A.); michele.cillo@unina.it (M.C.)

**Keywords:** Nodal, Cripto-1, proliferation, drug resistance, biomarker, therapeutic targets

## Abstract

The transforming growth factor beta (TGF-β) signaling is fundamental for correct embryonic development. However, alterations of this pathway have been correlated with oncogenesis, tumor progression and sustaining of cancer stem cells (CSCs). Cripto-1 (CR-1) and Nodal are two embryonic proteins involved in TGF-β signaling. Their expression is almost undetectable in terminally differentiated cells, but they are often re-expressed in tumor cells, especially in CSCs. Moreover, cancer cells that show high levels of CR-1 and/or Nodal display more aggressive phenotypes in vitro, while in vivo their expression correlates with a worse prognosis in several human cancers. The ability to target CSCs still represents an unmet medical need for the complete eradication of certain types of tumors. Given the prognostic role and the selective expression of CR-1 and Nodal on cancer cells, they represent archetypes for targeted therapy. The aim of this review is to clarify the role of CR-1 and Nodal in cancer stem populations and to summarize the current therapeutic strategy to target CSCs using monoclonal antibodies (mAbs) or other molecular tools to interfere with these two proteins.

## 1. Nodal and Cripto-1 Signal Cascade

The transforming growth factor β (TGF-β) signaling family plays a pivotal role in early vertebrate development, in inflammation and in host immunity [1]. In the earliest stage of embryo development, these secreted signaling molecules orchestrate the positioning and fate of different cells by creating morphogen gradients [2]. TGF-β superfamily members are highly conserved and share the same mechanism of action by binding to transmembrane serine/threonine kinase receptors, which in turn activate intracellular signal transducers [3]. As part of the TGF-β subfamily, the Nodal signals are critical for the induction of mesoderm and endoderm formation and for the specification of left-right axis asymmetry [4]. Nodal ligands are initially translated as precursor protein constituted by dimeric proproteins linked by disulfide bonds which are cleaved, generating active mature forms by extracellular proteases furin and PACE4 [5]. Nodal ligands bind to cell-surface serine/threonine kinase receptors known as TGF-β type I, also known as Activin receptor-like kinase4/7 (ALK4/7), and type II receptors (ActRIIA/ActRIIB) [6]. Ligand binding induces formation of heterotetramers constituted by two type I receptors and two type II receptors [7]. The type II receptor phosphorylates and activates the type I receptor which in turn phosphorylates and activates the type II [8]. This activation mediates the exposure of docking site for SMAD Anchor for Receptor Activation (SARA) in the endosomal compartment which recruits receptor-regulated SMAD proteins (R-SMADs) [9]. R-SMADs (SMAD1, 2, 3, 5, 8) are directly phosphorylated by ALK receptors and then associate with SMAD4, forming a heterotrimer that translocates to the nucleus and regulates target gene transcription in cooperation with various DNA-binding partners [10]. This signal cascade is tightly regulated also by Inhibitory SMADs (I-SMADS) that counteract the effect of R-SMAD antagonizing TGF-β signaling [11].

As described above, Nodal signal is transduced from the membrane to the nucleus through cooperation of proteins which activation is regulated by Nodal itself. Indeed, SMAD heterodimers bind to transcription factors that regulate Lefty genes transcription. Lefty proteins are antagonists of Nodal morphogens, switching off Nodal signal cascade and thus acting as negative feedback regulators [12].

Nevertheless, Nodal proteins show a different affinity to TGF-β receptors; it was demonstrated that the presence of binding proteins that act as partners enhances the signal cascade, improving the interactions between Nodal and its receptors. It was established that a fundamental obligatory co-receptor for the TGF-β family member Nodal is the cell surface glycosylphosphatidylinositol (GPI)-linked glycoprotein Cripto-1 (CR-1) [13]. Co-immunoprecipitation experiments show the interaction between Nodal and CR-1, supporting the idea that CR-1 acts as an obliged coreceptor for Nodal that potentiates its signal cascade [14,15,16]. However, evidence shows that Nodal has signal activity even in the absence of CR-1 interacting directly with its receptor [17]. CR-1, also known as Teratocarcinoma-derived growth factor1 (TDGF-1), was firstly identified in teratocarcinoma cells NTERA2 [18]. It belongs to the EGF-CFC (Cripto-1/FRL1/Cryptic) family that encodes a group of structurally related proteins that serve as important factors during early embryogenesis in vertebrates such as *Xenopus*, *Zebrafish*, mice, and humans [19]. Prior to gastrulation event, in the epiblast, CR-1 expression is symmetrical and uniform. Then, it forms an asymmetrical proximal-distal gradient, shifting caudally towards the region of the nascent primitive streak, regulating A-P patterning [20,21].

As other members of the EGF-CFC family, CR-1 contains an NH2-terminal signal peptide, a modified EGF-like region, a conserved cysteine-rich domain (CFC motif), and a short hydrophobic COOH-terminus that contains additional sequences for GPI cleavage and attachment that generate a biologically active soluble form [22]. Site-directed mutagenesis experiments showed that CR-1 directly interacts with ALK4 through its CFC domain and accommodates Nodal protein by interacting through its EGF-like motif. The binding between CR-1 and ALK4, and in particular the substrate-determining loop (L45) of ALK4, is fundamental for Nodal-induced SMAD2 activation. In fact, alterations of this loop are sufficient to redirect the Nodal signal from SMAD2 to SMAD1 activation through the ALK4/CR-1 complex [23]. In addition, Watanabe et al. showed that the GPI-signal sequence of CR-1 is necessary to induce optimum Nodal signaling in *trans* as well as in *cis*, demonstrating that both transmembrane and soluble CR-1 forms are active to induce Nodal signal cascade [24].

CR-1 has been shown to mediate signaling for other TGF-β ligands such as Activin and Xenopus Vg1 and its ortholog in mouse GDF1 [25]. In all cases, except for Activin, CR-1 acts as an enhancer of the signal cascade. Indeed, CR-1 can bind Activin in the presence of ActRII/ActRIIB, creating a complex that inhibits Activin-ALK4 interaction and blocking downstream signaling. This antagonistic behavior provides proof that CR-1 is involved in the regulation of TGF-β signal transduction and in cellular growth [26].

In addition to the regulator function of the TGF-β ligands signaling, CR-1 also plays a role in the Nodal independent signal cascade [27]. Several studies demonstrated the potential role of CR-1 in the progression of cancer, so it was speculated that this protein was involved in the regulation and/or activation of signal cascades that promote cell survival and proliferation. It was found that the oncogenic feature of CR-1 is due to its ability to activate the c-src/mitogen activated protein kinase (MAPK) and phosphatidylinositol 3-kinase (PI3K)/Protein Kinase B (AKT) signaling [28]. In fact, CR-1 may enhance the phosphorylation of MAPK and AKT in EpH-4 mouse mammary epithelial cells and MC3T3-E1 osteoblast cells that lack Nodal and ALK4 expression, respectively [29]. Moreover, the activation of these two signaling cascades is sustained by CR-1 interaction with its binding partner Glypican-1 [28]. This interaction may occur within plasma membrane lipid raft microdomains that play a key role in the activation of PI3K signaling by facilitating the interaction of tyrosine-protein kinase Src with specific PI3K isoforms [30].

Interestingly, subsequent experiments aimed at identifying novel CR-1 interacting partners enlighten the critical role of glucose-regulated protein 78 (GRP78) in strengthening CR-1 signaling. GRP78 is a member of the heat shock proteins and is mainly expressed in ER where it acts as a molecular chaperone [31]. Recently, the presence of GRP78 was demonstrated on the plasma membrane under stress conditions or ER stress [32,33]. CR-1 and GRP78 cooperate to promote pro-survival pathways responsible for chemo- and radio-resistance [34]. In particular, CR-1 is able to induce the expression of Survivin through the activation of the TAK-1/NF-kB pathway, and independently from the PI3K/AKT axis, another inductor of Survivin [35].

Finally, several studies suggest a potential role of the CR-1 signaling pathway with the Wnt/β-catenin/Lef-1 signaling pathway [36]. CR-1 regulates the Wnt/β-catenin pathway by interacting with Frizzled Receptor 7 (FZD7) and Disheveled 3 (DVL3) stabilizing DVL3 expression levels and enhancing downstream signal cascade [37]. In addition, CR-1 has been identified by microarray analysis as a primary target gene in the Wnt/β-catenin signaling pathway during embryonic development [38]. Furthermore, mouse and human CR-1 enhance signaling through the canonical Wnt/β-catenin signaling pathway by interacting with LRP5 and LRP6 co-receptors [39].

**Figure 1:**
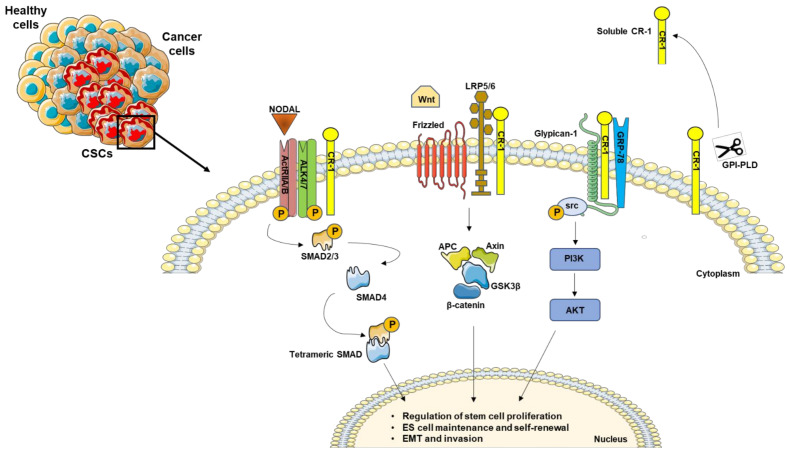
Schematic representation of Nodal/CR-1 signaling pathway in cancer stem cells. CR-1 acts as a cofactor for Nodal to activate downstream SMADs effectors. In the absence of Nodal, CR-1 can also signal in a non-canonical pathway through Frizzled and LRP4/5 complex, activating β-catenin signaling. In addition, CR-1 interacts with Glypican-1 and GRP78, triggering PIK3/AKT activation by Src phosphorylation. In addition, the GPI-specific phospholipase D (GPI-PLD) is able to cleave CR-1, generating a biological active soluble form.

Recently, an additional role of these signal cascades was demonstrated in the maintaining of embryonic stem cell pluripotency [40], and in the sustaining of the cancer stem cell (CSC) compartments (Figure 1), which is discussed in the next section.

## 2. The Role of Nodal and Cripto-1 in Cancer Stem Cells

In 1997, a paper published in Nature Medicine reported the identification of an Acute Myeloid Leukemia stem cell-like population that was able to induce a tumoral mass identical to the original one if transplanted in NOD/SCID mice. These cells were characterized by the phenotype CD34^+^/CD38^−^ and were called SCID Leukemia-initiating cells (SL-ICs) [41]. In the following decade, such populations were found in almost all types of solid tumors including breast [42], colon [43,44], pancreatic [45,46], thyroid cancer [47], melanoma [48], brain tumors [49,50], and many others [51,52,53]. Nowadays, it is well established in the research community that cancer is a heterogenous pathology and that within cancer cells, there is often a sub-population referred to as cancer stem cells (CSCs), that behaves like a stem population. As their healthy counterparts, CSCs possess an independent niche that protects them [54]. In addition to the self-renewal capacity, CSCs are usually slow-cycling [55]. For all these reasons, unfortunately, CSCs also display strong radio- and chemo-resistance, and they are thought to be the cancer initiator as well as the origin of relapses [56].

Another characteristic diffused in but not limited to CSCs is the re-activation of some embryogenic signaling pathways that are fundamental for “normal” stem cells [57]. This is often due to the aberrant expression of some key proteins involved in signaling pathways which are typically active in embryonic and/or stem cells. The re-activation of such pathways allows cancer cells to acquire more aggressive phenotype, accelerating epithelial–mesenchymal transition (EMT) hence invasiveness and metastasization.

Numerous studies have found CR-1 and/or Nodal constitutively overexpressed in various human cancers, where their high expression is strongly associated with a worse prognosis [58,59,60,61,62].

In 2010, Watanabe and colleagues identified, on the basis of CR-1 expression, two distinct populations of cells in human embryonal carcinomas. CR-1^high^ expressing cells displayed a more tumorigenic behavior compared to CR-1^low^ ones, both in vitro and in vivo [63]. The group also studied the promoter region of the TDGF1 gene. Interestingly, they found that Nanog and Oct4, two transcription factors (TFs) highly active in stem cells [64,65], bind CR-1 promoter and induce its transcription. This evidence was also confirmed by another group in the same cancer type [66]. However, silencing of CR-1 in these cells does not significantly alter the expression of Oct4 and Nanog, suggesting the existence of a Cripto-independent pathway that sustains the expression of these stem TFs. This is probably achieved by the activity of SMAD2/3 that can directly induce Nanog and Oct4 [63].

Several other findings strongly support the involvement of CR-1 in CSCs. In 2017, the results obtained by Watanabe and colleagues were replicated in esophageal squamous cell carcinoma (ESCC) cell lines [67]. Mouse-induced CSCs exposed to soluble CR-1, which compete with endogenous CR-1 for Nodal signaling, display a reduction in the self-renewal ability, as well as a decrease in the ability to migrate and form spheroids in vitro [68]. In a recent study, it was demonstrated that the expression of CR-1 in human CSCs from colorectal cancers (CRC) spheroids originated from patients is not stationary. On the contrary, the surface expression of CR-1 (as well as its secretion in the tumor microenvironment) tends to oscillate during spheroids culture. The authors not only elegantly demonstrated that this regulation is coordinated in the entire tumor mass, but also that CR-1-silenced cells generate smaller colonies in soft-agar assays. By using in vivo tetracycline inducible silencing of CR-1 they also found that *Cr-1* knockdown CSCs generates smaller tumor masses, with fewer CSCs and a dramatically reduced ability to generate metastasis compared to non-induced controls [69].

How does CR-1 regulate stemness? Although the detailed mechanism is still currently being investigated, there is an increasing amount of evidence unveiling CR-1′s role in this context. In hepatocellular carcinoma (HCC) cells, CR-1 binds and stabilizes DVL3, helped by the concomitant binding of the Frizzled receptor FZD7 and the transmembrane lipoprotein LRP6, resulting in a more sustained signal from the Wnt/β-catenin axis. Experiments performed in overexpression system (HEK293T) have confirmed the binding of CR-1 to LRP6 and LRP5, underlining the ability of CR-1 to affect Wnt/β-catenin signaling [39]. Moreover, in HCCs, it was highlighted for the first time the correlation between high levels of CR-1 expression and its gene hypomethylation. Supporting this evidence, the treatment of the HCC cell line Hep3B with 5-Azacytidine, a demethylating agent, enhanced CR-1 expression [37].

Nodal seems to play a similar role in regulating the delicate balance that exists between stemness and differentiation. Its role has been extensively investigated in normal or embryonic stem cells [70,71], as well as in tumors.

Several studies focusing on breast cancer (BC) found that the expression of Nodal strictly correlates with the expression of stem cell markers such as CD44 and CD133 and embryonic TFs such as Sox2, Oct4, and Nanog [72]. In particular, phosphorylation of SMAD2/3 following Nodal stimulation can directly induce the expression of Nanog, OCT4, and Sox2 to maintain the pluripotency state of these cells [72,73,74]. Coherently with this evidence, silencing of Nodal is associated with a reduction in these markers [72]. Similar results were also observed in undifferentiated testicular germ cell tumors [75] and in pancreatic CSCs. In the latter, the stem subpopulation is enriched in CD133 and shows a significantly higher expression of pluripotency-associated genes [76].

Analogously, the expression of Nodal is strongly augmented in CRC biopsies compared to normal adjacent tissues [77]. Moreover, purified CSCs (CD24^+^ and CD44^+^) from CRC cell lines also revealed an increase in Nodal transcript and protein compared to the non-stem population [77].

Very recently, Nodal was found to induce the expression of L1CAM and CXCR4 in hypoxic microenvironment, which is typical of the stem cell niches. The population identifiable by L1CAM^high^/CXCR4^high^ expression shows stem-like characteristics and is more tumorigenic and chemo-resistant. On the contrary, the depletion of these cells, as well as interfering with Nodal, restores the sensitivity towards chemotherapeutics such as 5-fluorouracil (5-FU) [78].

As for CR-1, although enormous improvements have been made in the past decade, the intricate interactome and the multiple biological roles of Nodal remain difficult to deeply investigate the molecular mechanism underlying its role in contributing to and maintaining stemness in CSCs.

## 3. Nodal and Cripto-1 as Theranostic Targets

The deciphering of the multiple genetic lesions that drive cancer spread is addressed to develop new therapeutic approaches that may be combined with traditional strategies to overcome malignancies.

Most of the anti-cancer treatments widely used today were developed prior to 1975. At the time, scientists and researchers wanted to exploit the emphasized characteristics of cancer cells by interfering with behaviors shared with their healthy counterparts. In the past few decades, the knowledge about the hallmarks of cancer allowed the scientific community to understand strengths and weaknesses of many neoplastic diseases. The studies about the molecular signature that sustain cancer growth reveal, in some cases, the dependence of certain human cancer cells on a gene and/or an oncogene that drives the transforming process. This situation has been termed oncogene addiction and represents an important weakness of cancer cells; indeed, these genes have an irreplaceable role in sustaining cancer growth [79].

On the other hand, chemoresistance is the “sword of Damocles” in cancer therapy as the new insights about the plasticity and the self-renewal capacity of CSCs define new scenarios that hinder the success of cancer therapies [55]. In the past few decades, the major challenge of most scientists has been to identify the unique profile that characterize the subpopulation of CSCs searching for antigens and biomarkers to be used as attractive targets [80]. Several studies have considered CR-1 and Nodal proteins as promising and useful prognostic as well as diagnostic biomarkers [81]. It is well established that CR-1 levels in sera of patients with non-small cell lung cancers [82], in testicular germ cell-tumors [83], in HCC [83], and in clear cell renal carcinomas [84] is correlated with poor disease outcome. Liu et al. [67] highlighted that CR-1^high^ cells exhibit highly invasive and metastatic phenotype in ESCC. In addition, CR-1^high^ cells in ESCC express high levels of stemness-related genes, demonstrating that CR-1 plays a fundamental role in the establishment of CSC niches. Soluble CR-1 is detected in plasma patients affected by BC and CRC. Recent evidence suggests that CR-1 might be a useful marker as it was found at an early stage of disease in patients with BC compared with benign lesion and healthy counterparts. With regards to CRC, CR-1 is clearly detectable in serum of patients affected by CRCs, even if it does not correlate with tumor size or staging/grading [85]. Furthermore, a higher positivity for CR-1 has been observed in healthy colon tissues from high-risk individuals (one or more cases of CRC among first-degree relatives) [86].

**Figure 2:**
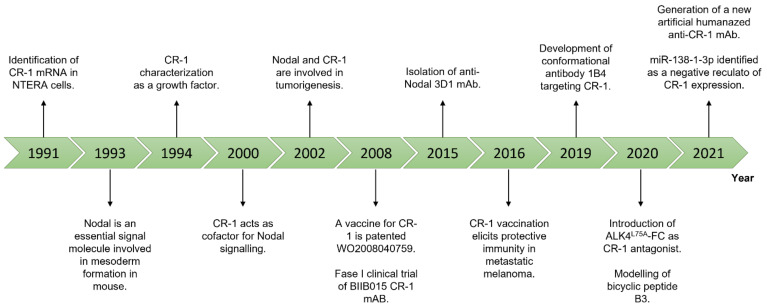
Timeline of the most relevant findings about Nodal and CR-1.

In this context, CR-1 and Nodal represent ideal candidates. A schematic representation of the most relevant findings about Nodal and CR-1 is provided in Figure 2.

What follows is a summary of therapeutic strategies that are currently, or might be, used.

### 3.1. Monoclonal Antibodies

This collection of evidence inspired many research groups to try to interfere with CR-1 function by using several molecular technologies. Monoclonal antibodies (mAbs) directed against several CR-1 domains were generated. They modulate CR-1 signal cascade inhibiting CR-1/Nodal transduction in transformed cells [87]. Focà et al. isolated a conformational mAb named 1B4 directed against the synthetic folded CFC (aa. 112−150) domain of the human protein, which antagonizes CR-1 signal cascade. They perform in vitro functional analysis of 1B4 mAb by using C8161 melanoma cell line and demonstrated that 1B4 is able to block and interfere with CR-1 signal cascade [88]. The antitumor effect of CR-1 mAb against EGF-like motif in vivo was potentiated in combination with cytotoxic drugs such as 5-FU in LS174T and MCF7 cells [89,90].

Moreover, a novel antagonist, ALK4^L75A^-Fc, constituted by a human Fc domain fused to a mutant ALK4 extracellular domain bearing a point mutation (L75A), interferes with CR-1/ALK4 interaction. ALK4^L75A^-Fc is effective against poorly differentiated triple negative BC cells altering stress response properties typical of breast CSCs [91].

Very recently, Ishii and colleagues generated and characterized a new humanized artificial Ab targeting CR-1 which shows high affinity for the recombinant human protein. In addition, this Ab is able to decrease the growth of cancer cells overexpressing CR-1 (GEO, NTERA, and T47D), with IC_50_ in the nM range, while having no effects on HEK293 cells (very low expression of CR-1) [92].

A novel and interesting approach to interfere and target CR-1 expressing cancer cells could be the usage of bispecific antibodies (BiAbs) in association with a T cell-based therapy. Briefly, BiAbs can bind from one side an epitope that is expressed on the target cells, while on the other side they can be recognized by synthetic agonistic receptor (SARs) of engineered cytotoxic T cells, thus activating the immune system. An in vitro study has already shown the feasibility of this approach by selecting CR-1 as the activator antigen for SAR-T cells [93].

As for Nodal-targeting Abs, an mAb named 3D1 was developed and well characterized. It is able not only to recognize and bind endogenous Nodal levels in different experimental settings (Western Blot, Flow Cytometry and ELISA), but it can also inhibit Nodal signaling by decreasing phosphorylation levels of SMAD2 and ERK1/2 while inducing p27. More interestingly, in vivo, the reduction in pSMAD2 levels is also appreciable in mouse xenografts of C8161 and A375SM human melanoma cell lines, with a sensitive decrease in tumor volume, when compared to a control IgG [94,95]. The biological effects of this Ab are due to the fact that the region of Nodal targeted by the 3D1 is the pre-helix loop and the H3 helix of Nodal, which is the fundamental helix that interacts with CR-1 [96].

Despite the abundance of such mAbs (especially targeting CR-1), only a phase I clinical trial (NCT00674947) started in 2008 with the aim to evaluate the safety and the maximal-tolerated dose of BIIB015 [97], an anti-CR-1 mAb that carries a payload made of DM4 (a microtubule disruptor). However, even if preclinical results obtained both in vitro and in vivo models and the trial ended in 2011, no results were published to date. This reflects a peculiar aspect of the interference with the TGF-β pathway in general—nearly the totality of the attempts made to target this pathway for cancer therapy have failed or showed only little improvement in survival, not justifying the adverse effects (especially cardiac) [98,99]. One of the reasons may reside in the fact that TGF-β signaling is extremely articulated and regulated at multiple levels.

### 3.2. Oligonucleotides-Based Therapies: Antisense Oligos, miRNAs, and circRNAs

Through the usage of oligonucleotides, it is possible to modulate gene expression from single cells level to entire organisms. The increasing advances in oligonucleotides optimization and their delivery tools, as well as the approval of several oligonucleotide-based drugs, enables, at least in theory, the modulation of cancer vital genes such as *Cr-1* and *Nodal*. Different miRNAs were found altered in several human cancers.

The first attempts to interfere with CR-1 expression using antisense oligonucleotides date back to the 1990s in CRC cells. The reductions in CR-1 protein levels were associated with a global reduction in tumorigenicity, such as decreased growth potential, reduced number of colonies in soft agar assays or smaller tumor mass induction in mouse xenografts, and a regained sensitivity towards some chemotherapeutics such as 5-FU [100,101,102,103]. Interestingly, these anti-tumoral effects were synergistically enhanced by using anti-EGFR mAb [104]. Similar results were obtained in ovarian cancer [105] and human embryonal carcinoma cell line NTERA2 [106].

Besides antisense oligonucleotides, an important regulating role is played by miRNA. In nasopharyngeal carcinoma (NPC), miR-138-1-3p has been inversely correlated with the expression of CR-1. In fact, radioresistant NPC cell lines display a reduction in miR-138-1-3p together with an increased CR-1 expression [107]. Inducing the expression of miR-138-1-3p has been proven to reduce the activation of the JAK2/STAT3 pathway, which is sustained, among others, by the GRP78-dependent soluble CR-1 signaling, resulting in increased proliferation and survival [108]. For example, Sun and colleagues demonstrated that the miR-15b is able to bind the 3′ UTR of Cripto-1 with its consequent downregulation. Tumors such as gliomas often downregulate miR-15b. On the contrary, transfection of this miRNA leads to cell cycle arrest and apoptosis through the regulation of CR-1 levels [109]. The same trend was also observed in non-small cell lung cancer (NSCLC) with miR-15a-16 [110].

Very recently, in CRC spheroids, two circular RNA (circRNA) were identified as upregulated in CSCs: circ_0066631 and circ_0082096. These two circRNA were computationally predicted to negatively regulate five miRNAs involved in the maintaining of stemness by downregulating, in turn, the expression levels of SMAD2 and ALK7, key proteins in CR-1 and Nodal signaling [111].

### 3.3. Small Molecule Inhibitors

Some small molecules and small peptides have been generated or identified in order to interfere especially with CR-1-mediated signaling.

A bicyclic peptide named B3 was modelled to interfere with CR-1 and ALK4 interaction inhibiting signal cascade in vitro. This small molecule is a CFC domain analogue and is directly cytotoxic for CR-1 positive NTERA cells at nanomolar concentrations (in vitro) [90].

Alantolactone is a natural compound derived from plants and it was revealed to be an inhibitor of CR-1 signaling because it interferes with the binding between CR-1 and ActRIIA. Its antiproliferative effects were tested on human colon adenocarcinoma cell line HCT-8, where it was able to reduce Activin signaling through the suppression of SMAD3 phosphorylation while displaying no effects on human normal liver cell line L02 [112].

Lastly, while not being a direct Nodal or CR-1 inhibitor, the chemical compound named SB-431542 is able to suppress endogenous Activin signaling through the interference with ALK4/7 receptors, which are crucial for the CR-1-mediated Nodal signaling [113].

However, it must be noted that the above-described molecules are, at least to date, not included in any (pre)clinical trial.

### 3.4. Cancer Vaccines

Immune response plays an important role in detecting cancer antigens and eliciting immune response against neoplastic cells. Cancer vaccines have been an appealing strategy to overcome tumor resistance to conventional therapies [114]. As for other cancer vaccines, the basic idea underlying CR-1 vaccination is to induce the production of Abs that are able to block CR-1 and Nodal signaling in cancer (stem) cells.

In 2008, the first CR-1 vaccine was patented (patent number WO2008/040759Al) [115]. The aim of this study was to stimulate antibody-based immune response to CR-1 enriched-cells by using a preventive or a therapeutic immunization. To this purpose, the authors generated a vaccine directed against CR-1 engineering several chimeric CR-1 peptides by adding to CR-1 wild-type protein, a foreign peptide that is recognized by T helper lymphocytes. Indeed, they exploited the pan DR-binding epitope (PADRE) to maximize T cell immune response by modifying CR-1, preserving its three-dimensional structure. Successfully, twelve CR-1 molecules were identified as containing PADRE (CR-1 Autovac), which represent an interesting tool of investigation.

Further investigations demonstrate that human CR-1 was an attractive target to stimulate humoral immune response by using vaccines [115,116]. CR-1-encoding DNA vaccines were used to stimulate humoral response in C57Bl/6 mice. After vaccination, CD8^+^ T cells isolated from the spleen of a CR-1-vaccinated mouse secreted significant amounts of IFNγ in response to stimulation with B16F10 melanoma cells that express high levels of CR-1 protein. These results demonstrate that CR-1 immunization induces CR-1-specific cytotoxic CD8^+^ T cells with the ability to recognize highly aggressive melanoma cells. This phenomenon was tested also by challenging C57Bl/6 mice with B16F10 melanoma cells. Indeed, immunized mice presented a significant delay in tumors growth and in the number of metastatic lung foci compared to the control empty vector immunized mice.

Afterwards, Witt et al. [116] demonstrated that this vaccine is also applicable to BALB-neuT mice that represent a dynamic model of BC. Spheroid-cultured Tubo cells isolated by BALB-neuT primary tumors develop a cancer stem cell signature characterized by high CR-1 expression levels. DNA vaccination induces a CR-1-specific humoral response, eliciting ADCC by NK cells, macrophages, and neutrophils. The therapeutic efficacy of CR-1 vaccine was confirmed by the reduction in lung metastatic loci in vaccinated BALB-neuT mice respective to the control counterparts.

In the past year, a global effort has been made to optimize the mRNA vaccination technology so that it can be safer and suitable for large-scale production. The possibility to deliver the mRNA through liposomes, using a variety of administration routes, is nowadays concrete and has been proven efficient. Such technology could be potentially used not only to immunize against viral diseases, but also in the field of cancer vaccination [117].

## 4. Conclusions

The ability of cancer cells to take advantage of pro-survival stimuli and resist the organism’s attempts to eradicate them is a notion that research community acquired decades ago. Dozens of these pathways have been identified so far, including p53, NF-kB, PI3K, and MAPK as well as several growth factors and cell cycle regulatory pathways. Among these, a remarkable body of literature has evidenced the deep involvement of TGF-β family signaling in cancer. As key proteins in this pathway, Nodal and CR-1 represent ideal candidates for a “two-level” targeted therapy. The first level considers that they are both absent and low-expressed in adult tissues, which should reduce the adverse effects correlated to the interference with the two proteins. The second level is represented by the frequent overexpression of Nodal and/or CR-1 in the CSC compartments of several solid tumors, potentially allowing treatments to hit the most difficult and less targetable sub-population, which is thought to be the one that thwarts the complete tumor eradication. In addition, CR-1 and Nodal overexpression is correlated with a worse prognosis in several human cancers. For all these considerations, CR-1 and Nodal have the high potential to be both diagnostic and therapeutic targets consistent with the so-called “theranostic” approach.

The availability of different tools and strategies to target both CR-1 and Nodal render this an exciting field of study. mAbs are, so far, the best candidates due to their high specificity for the targets, allowing them to precisely hit cancer cells while simultaneously reducing the adverse effects. These mAbs can be produced by the modern biotech industry, or ideally an immune response can be elicited through cancer vaccines. These new therapeutic tools may be clinically effective alone or in combination with the conventional radio- or chemo-therapies to maximize the effects, targeting all the sub-populations of which a tumor is composed.

However, an important aspect should be considered: CR-1 and Nodal (or in general TGF-β signaling) are involved in maintaining the stemness of physiological stem cells. To the best of our knowledge, no studies have been conducted to address the impact that such treatments could have on the healthy stem cell compartments.

## Data Availability

Not applicable.
